# Elevated Level of CD4^+^ T Cell Immune Activation in Acutely HIV-1-Infected Stage Associates With Increased IL-2 Production and Cycling Expression, and Subsequent CD4^+^ T Cell Preservation

**DOI:** 10.3389/fimmu.2018.00616

**Published:** 2018-03-27

**Authors:** Huan Xia, Wei Jiang, Xin Zhang, Ling Qin, Bin Su, Zhen Li, Jianping Sun, Yonghong Zhang, Tong Zhang, Xiaofan Lu, Hao Wu

**Affiliations:** ^1^Beijing Key Laboratory for HIV/AIDS Research, Center for Infectious Diseases, Beijing You’an Hospital, Capital Medical University, Beijing, China; ^2^Department of Microbiology and Immunology, Medical University of South Carolina, Charleston, SC, United States; ^3^Division of Infectious Diseases, Department of Medicine, Medical University of South Carolina, Charleston, SC, United States; ^4^Biomarkers of Infection Related Diseases, Beijing Key Laboratory, Beijing You’an Hospital, Capital Medical University, Beijing, China

**Keywords:** acute HIV-1 infection, T cell immune activation, CD4^+^ T cell, immune exhaustion molecules, T cell preservation

## Abstract

Persistent immune activation is a striking consequence of HIV-1 infection and a driving force of CD4^+^ T cell depletion and AIDS events during chronic infection. High level of T cell immune activation associates with antiretroviral therapy (ART)-treated clinical outcomes in chronically HIV-1-infected patients. However, the role of T cell activation during acute infection stage in subsequent CD4^+^ T cell decline in the absence of ART treatment is unknown. In this study, we enrolled 26 acutely HIV-1-infected patients in the absence of ART treatment from a prospective acute HIV-1 infection cohort in Beijing (PRIMO). A comprehensive analysis of CD4^+^ and CD8^+^ T cell immune activation during acute infection stage and the clinical outcomes was studied. We found that patients with higher level of CD4^+^ T cell activation (%CD38^+^HLA-DR^+^CD4^+^ T cells) exhibited more effective function (%IL-2 production and %ki67 expression) in CD4^+^ T cells compared to those from patients without increased T cell activation at the acute phase. Direct correlations were observed between CD4^+^ T cell activation and the percentages of IL-2-producing or ki67-expressing CD4^+^ T cells in patients at the acute phase of infection. Importantly, the increased levels of CD4^+^ T cell immune activation, IL-2 production, and cycling expression during acute infection were associated with less decline of CD4^+^ T cell after 2 years of infection. However, immune exhaustion molecules in acute infection, including CD160, T cell immunoglobulin and ITIM domain, programmed cell death protein 1, and T cell immunoglobulin and mucin 3, were not associated with the CD4^+^ T cell depletion. These significant associations of CD4^+^ T cell activation were not demonstrable for CD8^+^ T cell activation at the acute phase. Taken together, our observations provide new insight into the possible role of T cell activation in preventing CD4^+^ T cell depletion during acute HIV-1 infection.

## Introduction

Chronic immune activation and inflammation have been known to be a hallmark of HIV-1 infection. They act as independent predictors of disease progression and are also considered as main driving force of non-AIDS morbidity and mortality (1–3). Inflammation refers to cytokines released by macrophages upon antigen stimulation, whereas immune activation describes cellular activation in response to stimuli, reflected by increased expression of activation markers on T cells and antigen presenting cells (e.g., monocytes) (4). Double expression of CD38 and HLA-DR on T cells often represents the level of T cell immune activation (5). Several factors contribute to immune activation in HIV-1-infected individuals, such as HIV-1 replication, microbial products leaking from damaged gut mucosa, imbalance of Th17/Treg, and opportunistic infections with other virus or bacteria (6).

To date, most studies on T cell immune activation in HIV-1 infection focus on the chronic infection stage. Growing evidence suggests that persistent T cell immune activation during chronic stage contributes to the progressive CD4^+^ T cell depletion and immune perturbations (7–9). Elevated residual T cell immune activation is still observed despite successful antiretroviral therapy (ART) (10, 11). High activated CD8^+^ T cells in HIV-1 chronic infection stage is a strong predictor for shorter survival, faster decline of CD4^+^ T cells, and poor CD4^+^ T cell recovery under ART (1, 12). Notably, T cell immune activation has occurred during HIV-1 acute infection phase, and its set point is also established in this phase (2). The level of CD4^+^ and CD8^+^ T cell immune activation stabilizes for at least 48 weeks after reaching an immune activation set point (2). The set point of T cell immune activation predicts subsequent disease progression if ART is not initiated (2). The VISCONTI study (Viro-Immunological Sustained CONtrol after Treatment Interruption) reported that CD4^+^ and CD8^+^ T cell activation were very weak in posttreatment HIV-1 controllers during the period of viral control without therapy (13). However, unlike the consensus on the deleterious effects of sustained immune activation in chronically infected period, beneficial of T cell immune activation in acute infection were also observed (14, 15). Paul Volberding et al. illustrated that higher percentages of activated CD4^+^ T cells in early HIV-1 infection were associated with greater likelihood of virologic suppression upon treatment interruption (14). The study by Ndhlovu et al. reported that HIV-1-specific CD8^+^ T cell activation in acute phase were beneficial in controlling viremia (15). These evidences indicate a protective role of activated T cells in controlling viral replication and disease progression during acute HIV-1 infection. However, the role T cell activation in the acute phase in CD4^+^ T cell depletion in the absence of ART treatment is unknown.

Due to the controversial opinions on the role of T cell immune activation in HIV-1 acute infection, here we analyzed the association between the level of T cell immune activation in different disease stages and clinical outcomes by using samples from our HIV-1 acute infection cohort (PRIMO). We aimed to further study the different role of T cell immune activation in HIV-1 acute and chronic infection stages and the possible mechanisms involved.

## Materials and Methods

### Study Participants

Study subjects in this study were enrolled from Beijing PRIMO Cohort, high-risk men who have sex with men (MSM) cohort for screening HIV-1 acutely infected individuals at Beijing You’an Hospital (16). MSM who have high-risk behaviors were enrolled in this cohort, and were followed up for every 2 months to detect HIV-1 antibody. Acute infection was defined as a positive result of HIV-1 RNA but a negative or indeterminate result of HIV-1 antibody. Once acute infection was identified, patients were followed up at 1, 2, 4, 8, and 12 weeks, and then every 3 months for CD4^+^ T cell count and viral load measurement and syphilis status monitoring. Chronic infection was defined as 6 months after diagnosis of acute infection. Blood samples were collected at each time point, peripheral blood mononuclear cells (PBMCs) and plasma were isolated and cryopreserved.

All HIV-1-infected participants provided written informed consents according to the declaration of Helsinki. All relevant experiments have been approved by the Beijing You’an Hospital Research Ethics Committee. The methods applied were carried out in accordance with the approved guidelines.

A total of 26 HIV-1 acutely infected individuals (AHIs) were participated in this study. Samples of subsequently chronic infection stage of these AHIs were also collected and analyzed. Basic information of study subjects was reported in Table 1. No individual accepted ART. The progression of acute HIV-1 infection can be depicted as six discrete stages as proposed by Fiebig et al. (17, 18). These 26 AHIs were at Fiebig stage III–VI. 21 age-matched male MSM healthy donors (HDs) were included as controls.

**Table 1:** Patient characteristics.

Patient ID	Estimate infection days	CD4 count (cells/μl)		Viral load^a^ (copies/ml)	Days from the initial positive point to CD4 <300 cells/μl
				
		Acute stage	Chronic stage		
1	37	571	243	–	598
2	75	429	244	4,880	431
3	40	636	254	22,200	427
4	40	508	97	–	528
5	65	498	230	–	370
6	60	658	209	–	433
7	80	533	279	–	731
8	52	504	225	–	534
9	41	683	283	–	243
10	34	482	152	–	251
11	94	496	211	–	258
12	38	478	233	–	174
13	65	673	309	1,610	392
14	68	599	564	–	>2 years
15	56	419	469	–	>2 years
16	50	665	419	–	>2 years
17	45	659	490	–	>2 years
18	41	629	579	2380	605
19	79	688	251	–	175
20	75	498	210	258,000	323
21	34	500	241	–	344
22	92	550	208	–	>2 years
23	85	755	511	3,280	>2 years
24	78	496	537	–	>2 years
25	54	680	420	–	>2 years
26	41	600	466	–	>2 years

*^a^Plasma viral load were available for only six patients (acute infection stage)*.

*–, not available*.

### Flow Cytometry Analysis of T Cell Activation, Exhaustion, and Cycling Expression

Cryopreserved PBMCs were first stained with fixable viability stain 510 (BD Biosciences, San Diego, CA, USA) for 15 min to exclude dead cells, then washed, and surface stained with anti-CD3-BV650 (BD Biosciences), anti-CD4-AF700 (Biolegend, San Diego, CA, USA), anti-CD8-Percp-Cy5.5 (BD Biosciences), anti-CD38-BV786 (BD Biosciences), anti-HLA-DR-Pacific blue (Biolegend), anti-CD160-AF488 (eBioscience San Diego, CA, USA), anti-PD-1-BV605 (Biolegend), anti-TIGIT-APC (eBioscience), and anti-TIM-3-PE (R&D Systems, Inc., USA) at room temperature for 20 min. Cells were permeabilized and fixed with intracellular staining reagents according to the manufacturer’s instructions (eBioscience) before intracellular staining with anti-ki67-PE-Cy7 (Biolegend). Samples were then washed before acquisition and analysis on a BD LSRFortessa flow cytometer instrument with Diva software (BD Biosciences). Relative isotype controls or fluorescence minus one samples were prepared to facilitate gating. Data were analyzed using Flowjo Software version 10 (Tree Star Inc., Ashland, OR, USA).

### Intracellular Cytokine Staining

Peripheral blood mononuclear cells were stimulated by leukocyte activation cocktail with BD GolgiPlug (BD Biosciences) in an incubator at 37°C with 5% CO_2_ for 6 h, the leukocyte activation cocktail contained phorbol 12-myristate-13-acetate (PMA), ionomycin, and brefeldin A. After stimulation, cells were washed and stained with surface phenotype panel against CD3, CD4, and CD8 for 20 min at room temperature in the dark. After permeabilization and fixation, cells were intracellularly stained with anti-IFN-γ-BV605, anti-IL-2-APC, and anti-TNF-α-PE-Cy7 (all from Biolegend). Data were acquired and analyzed as described above.

### HIV-1 Viral Load and CD4^+^ T Cell Count Measurements

Plasma HIV-1 viral load (pVL) was quantified using automated RealTime PCR-based *m*2000 system (Abbott Molecular Inc., Des Plaines, IL, USA), with a detection limit of 40 copies/ml. CD4^+^ T cell absolute count were determined in fresh whole blood by using Trucount Tubes and CD45/CD3/CD4/CD8 four-color antibody (BD Biosciences) according to the manufacturer’s protocol.

### Statistical Analysis

Statistical analysis and graphical presentation were performed using GraphPad Prism version 5 software (GraphPad Software, San Diego, CA, USA). Data are expressed as median values and analyzed by the use of Mann–Whitney *U* test. Correlations between variables were estimated with Spearman’s rank correlation test. Survival analysis were generated from the log-rank test. All tests were two-tailed and *p* value < 0.05 was considered statistically significant.

## Results

### High Level of CD4^+^ T Cell Immune Activation in Acute HIV-1 Infection Was Associated With Subsequently Slow CD4^+^ T Cell Decline

**Figure 1:**
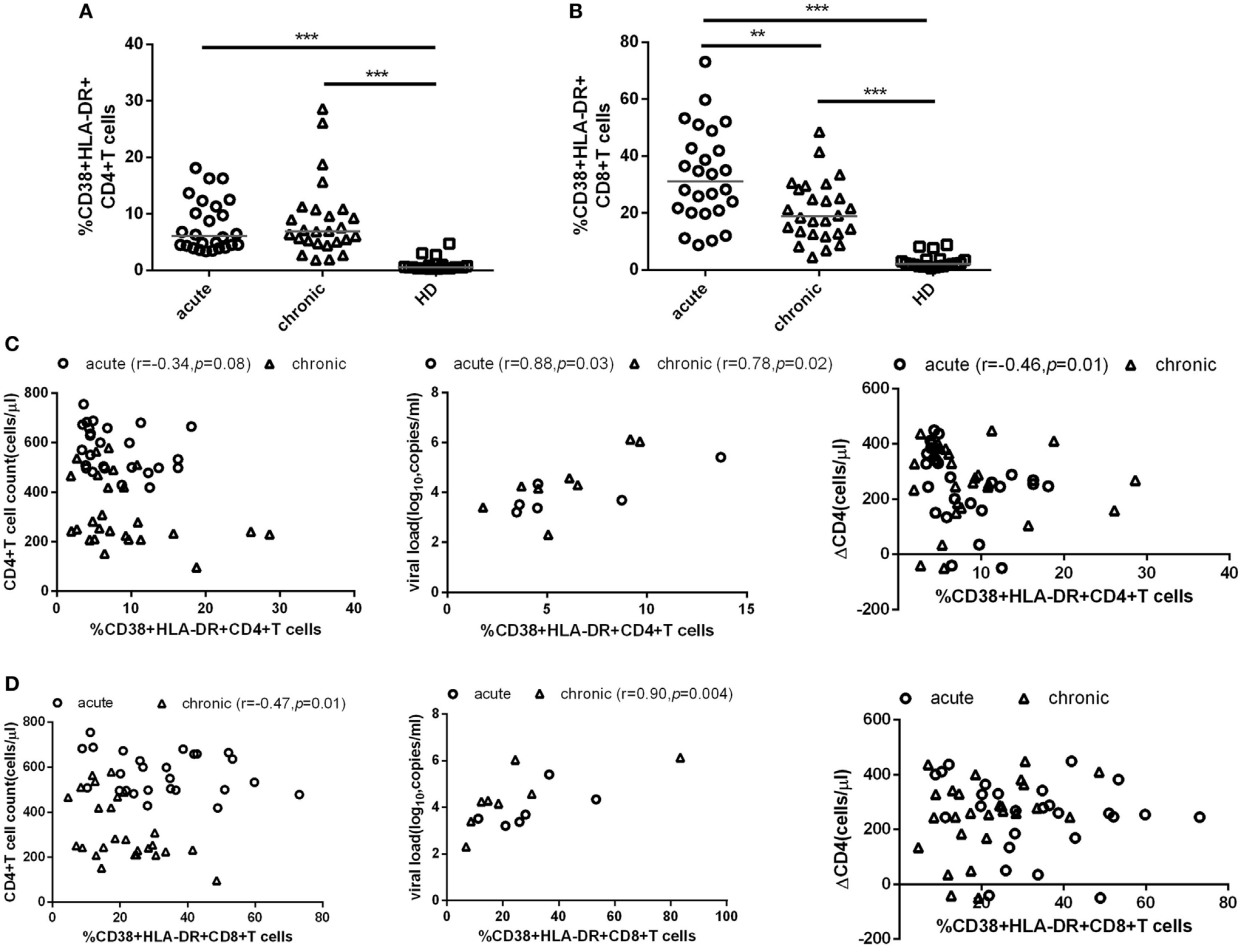
CD4^+^ and CD8^+^ T cell activation in HIV-1 acute and chronic infection stage. Comparisons of the level of CD4^+^ T **(A)** and CD8^+^ T **(B)** cell immune activation among healthy donors (HDs, □), HIV-1 acute (AHIs, ○) and chronic (chronic HIV-1 infection stage, Δ) infection stage. **(C)** Correlation between CD4^+^ T cell immune activation and CD4^+^ T cell count (*left*), viral load (*middle*), and CD4^+^ T cell depletion from acute stage to observation end-point in chronic stage (ΔCD4, *right*). **(D)** Correlation between CD8^+^ T cell immune activation and CD4^+^ T cell count (*left*), viral load (*middle*), and ΔCD4 (*right*). Significance of difference was calculated by Mann–Whitney *U* test. Correlation analysis was performed by Spearman’s rank test. Horizontal lines indicate median values (*p < 0.05, **p < 0.01, ***p < 0.001).

To investigate the role of T cell immune activation in the CD4^+^ T cell depletion during HIV-1 acute infection stage, we first compared the level of T cell immune activation between acute and chronic stage with paired HIV-1-infected patients’ samples. Here, we used the coexpression of CD38 and HLA-DR on the surface of CD4^+^ or CD8^+^ T cells to represent the level of T cell activation. As expected, the level of CD4^+^ and CD8^+^ T cell activation was significantly higher in AHIs and subsequently chronic HIV-1 infection stage (CHIs) compared with HDs (all *p* < 0.001, Figures 1A,B); however, no significant differences were found between AHIs and CHIs in CD4^+^ T cell immune activation (Figure 1A). Higher CD8^+^ T cell immune activation was observed in AHIs compared with CHIs (*p* < 0.01, Figure 1B).

Next, the relationship between the level of T cell immune activation and the variables of disease progression, e.g., CD4^+^ T cell count, pVL, and CD4^+^ T cell decline (ΔCD4) were assessed. ΔCD4 was defined as the change of CD4^+^ T cell count in 2 years after seroconversion. Consistent with other studies, during acute infection stage, the percentages of CD38 and HLA-DR coexpression on CD4^+^ T cells was negatively correlated with CD4^+^ T cell count (*r* = −0.34, *p* = 0.08, Figure 1C *left*) and positively correlated with pVL (*r* = 0.88, *p* = 0.03, Figure 1C *middle*). Interestingly, the higher level of CD4^+^ T cell activation in AHIs was correlated with less decline of CD4^+^ T cell count after 2 years of infection without ART (*r* = −0.46, *p* = 0.01, Figure 1C *right*). However, the same correlations between CD8^+^ T cell immune activation level and the disease progression marker (CD4^+^ T cell decline, CD4^+^ T cell count or pVL) were not found in AHIs (Figure 1D). Therefore, the level of CD4^+^ T cell immune activation in HIV-1 acute infection stage may predict the disease progression.

**Figure 2:**
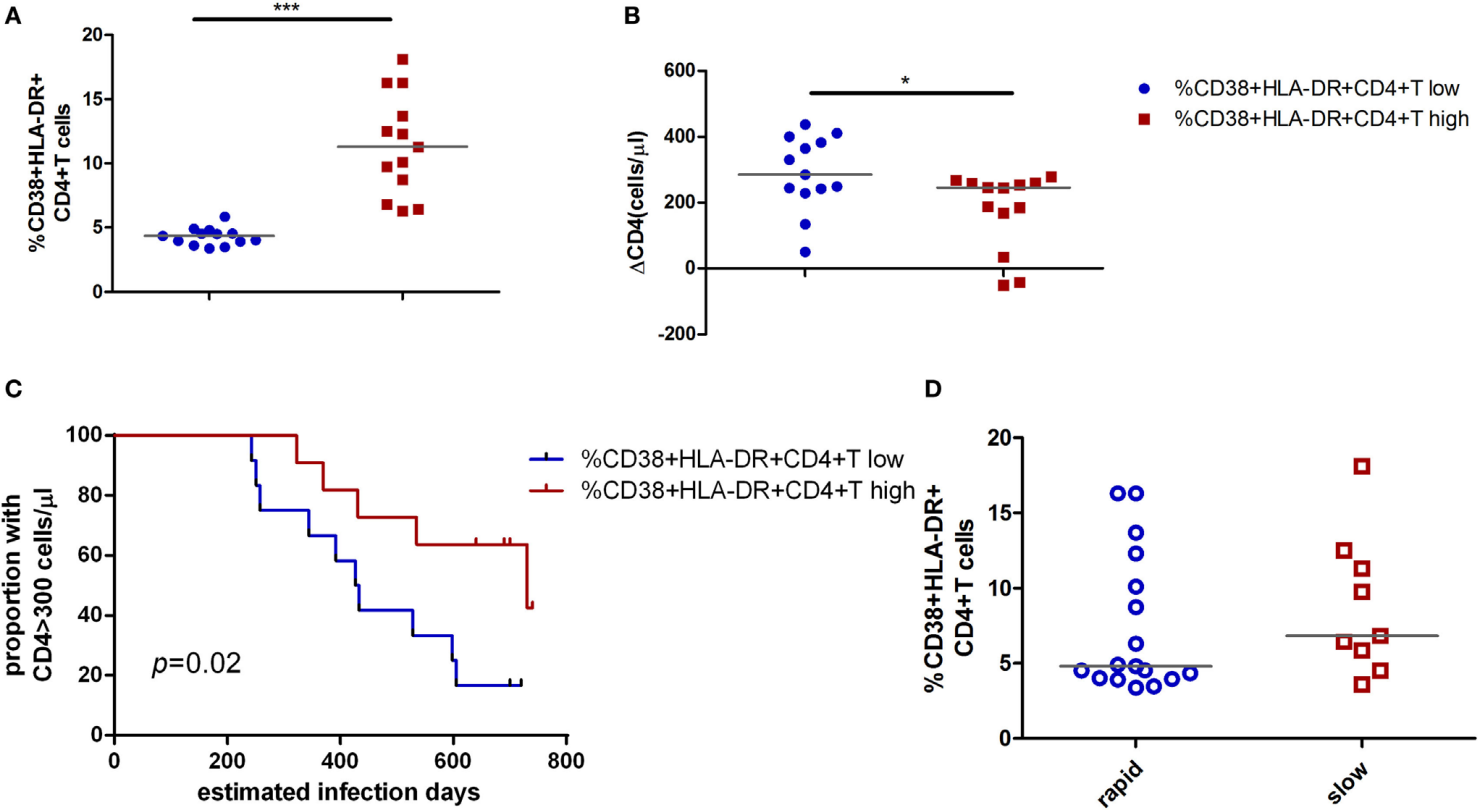
Impact of CD4^+^ T cell activation in acute HIV-1 infection on clinical outcomes. Comparison of the level of CD4^+^ T cell activation **(A)** and ΔCD4 **(B)** between CD4^+^ T cell activation high (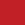) and low (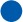) groups in acute infection stage. **(C)** Kaplan–Meier curves with univariate analyzes (log-rank) were performed to evaluate the effects of CD4^+^ T cell immune activation during acute HIV-1 infection stage on CD4^+^ T cell depletion. Endpoints were defined as CD4^+^ T cell count <300 cells/μl. Blue line, low CD4^+^ T cell activation; red line, high CD4^+^ T cell activation. **(D)** Comparison of the level of CD4^+^ T cell activation between rapid (○) and slow (□) progressors. Rapid progressors: CD4^+^ T cells drop to less than 300 cells/μl in 2 years after acute infection. Slow progressors: CD4^+^ T cells maintain to more than 500 cells/μl in 2 years after acute infection. Significance of difference was calculated by Mann–Whitney *U* test. Horizontal lines indicate median values (*p < 0.05, **p < 0.01, ***p < 0.001).

To further testify this possibility, all patients were classified into high or low activation groups based on the median values of CD4^+^ T cell activation (median value: 6.08%). Individuals in these two groups were not found significant difference in age, gender, and case history. The CD4^+^ T cell immune activation in these two groups was significant difference (*p* < 0.001, Figure 2A). ΔCD4 in CD4^+^ T immune activation high group was significantly lower than that in the low group (*p* < 0.05, Figure 2B). In the survival analysis, the low level of CD4^+^ T cell immune activation were significantly associated with faster time to the decrease of CD4^+^ T cell count below 300 cells/μl in chronic infection stage (*p* = 0.02, Figure 2C). Because CD4^+^ T cell activation at the acute phase associated with CD4^+^ T cell depletion at the chronic phase, we hypothesize that the CD4^+^ T cell immune activation in acute infection stage may play a protective role in the loss of CD4^+^ T cells during disease progression. To further testify whether CD4^+^ T cell immune activation in acute infection stage influences on the CD4^+^ T cell decline, patients were divided into rapid progressors (RPs, *n* = 17) and slow progressors (SPs, *n* = 9). RPs: change of CD4^+^ T cell count from baseline to the time point when their CD4^+^ T cell count dropped below 300 cells/μl; SPs: changes of CD4^+^ T cell count from baseline to 2 years of postinfection were not dropped below 300 cells/μl. Both RPs and SPs had same CD4^+^ T cell level at the blood collection time point in acutely infected stage in this study. Our results showed that SPs indeed had higher level of CD4^+^ T cell immune activation than RPs, although no significant difference between SPs and RPs (Figure 2D).

### CD4^+^ T Cell Cycling Expression During Acute HIV-1 Infection Is Correlated With CD4^+^ T Cell Immune Activation and CD4^+^ T Cell Decline Over Time

**Figure 3:**
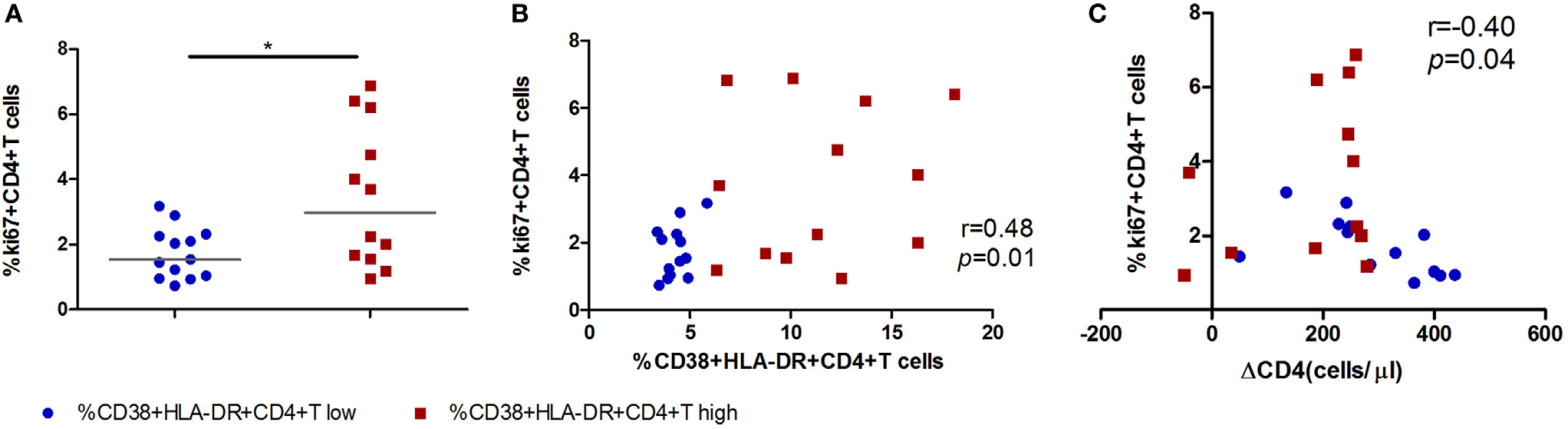
High CD4^+^ T cell immune activation is associated with greater cycling expression in CD4^+^ T cells in acutely infected patients. **(A)** Comparison of ki67 expression in CD4^+^ T cells in the high T cell activation (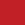) and low activation groups (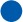). Correlation analyzes of ki67 expression and the level of CD4^+^ T cell activation **(B)** and ΔCD4 **(C)** in acute infection stage. Significance of difference was calculated by Mann–Whitney *U* test. Correlation analysis was performed by Spearman’s rank test. Horizontal lines indicate median values (*p < 0.05, **p < 0.01, ***p < 0.001).

To investigate the biologic function of CD4^+^ T cell immune activation in the acute infection stage in CD4^+^ T cell decline, we first analyzed the relationship between the proliferation capacity of CD4^+^ T cells and CD4^+^ T cell immune activation or the CD4^+^ T cell decline in AHIs. The expression of ki67 in CD4^+^ T cells represents the proliferation capacity (9, 19). We found that high CD4^+^ T cell immune activation groups had higher proliferation capacity than that in the low activation group (*p* < 0.05, Figure 3A). A direct correlation was observed between the percentages of ki67 in CD4^+^ T cells and the levels of CD4^+^ T cell activation (*r* = 0.48, *p* = 0.01, Figure 3B), and an inverse correlation was observed between the percentages of ki67 in CD4^+^ T cells and CD4^+^ T cell depletion in AHIs (*r* = −0.40, *p* = 0.04, Figure 3C). These findings suggest that the turnover of T cells is maintained during acute HIV-1 infection, implying that an effective proliferation response at the acute phase may contribute to the complement for CD4^+^ T cell depletion in the natural disease progression without ART.

### Immune Exhaustion in HIV-1 Acute Infection Stage Was Not Associated With CD4^+^ T Cell Decline

**Figure 4:**
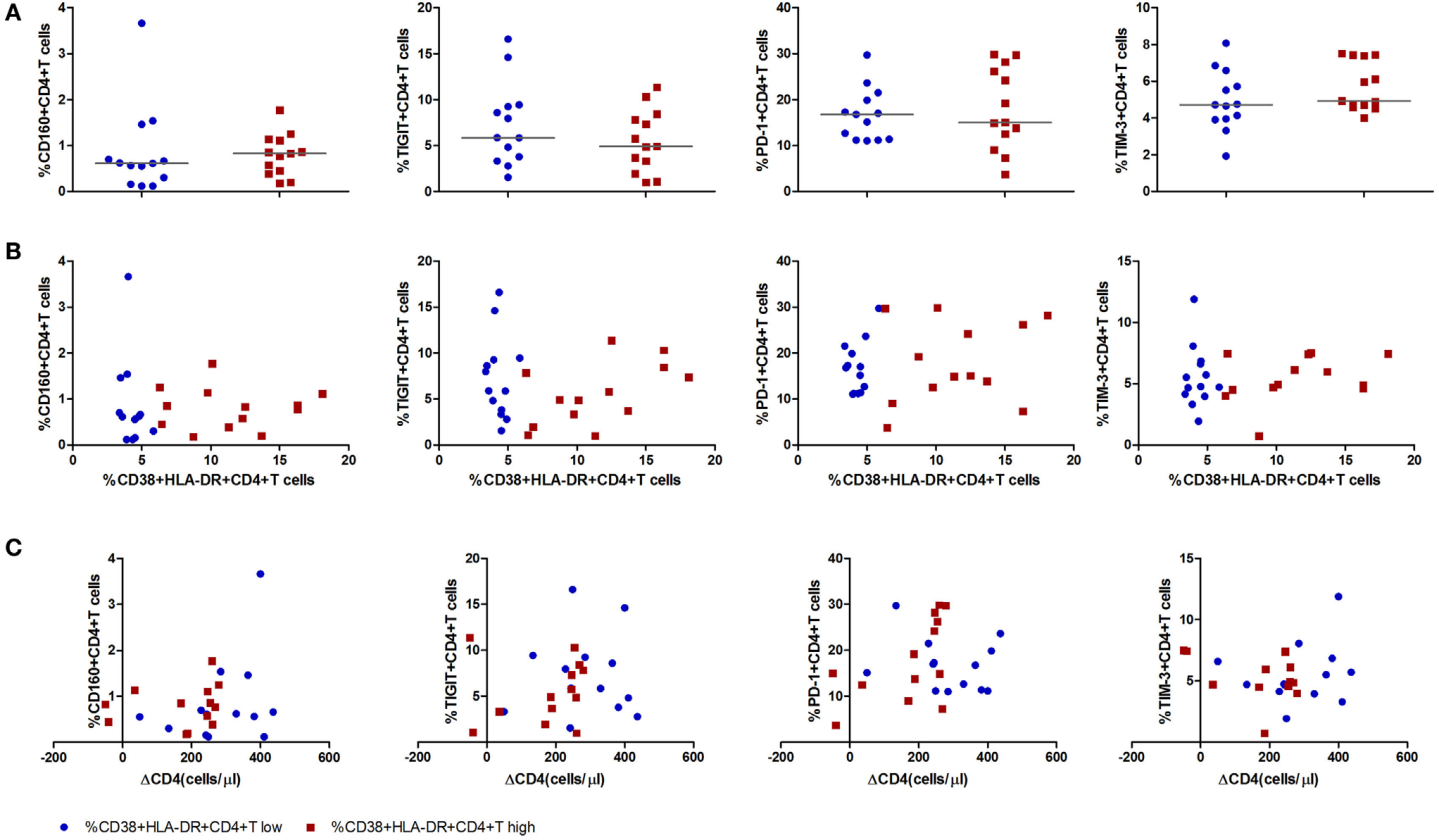
Expression of inhibitory molecules on CD4^+^ T cells in acute HIV-1 infection stage and their correlations with the level of CD4^+^ T cell activation and CD4^+^ T cell depletion. **(A)** Comparisons of the expression level of inhibitory molecules CD160, T cell immunoglobulin and ITIM domain, programmed cell death protein 1, and T cell immunoglobulin and mucin 3 on CD4^+^ T cell activation high (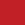) and low (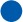) groups in acutely infected patients. Correlation analysis between inhibitory molecules and the level of CD4^+^ T cell activation **(B)** and ΔCD4 **(C)**. Significance of difference was calculated by Mann–Whitney *U* test. Correlation analysis was performed by Spearman’s rank test. Horizontal lines indicate median values.

Immune exhaustion was measured by expression of CD160, T cell immunoglobulin and ITIM domain (TIGIT), programmed cell death protein 1 (PD-1), T cell immunoglobulin and mucin 3 (TIM-3) on CD4^+^ T cells. The frequencies of CD4^+^ T cells expressing CD160, TIGIT, PD-1, TIM-3 were similar between the CD4^+^ T cell immune activation high and low groups (Figure 4A). No associations were observed between any one of the immune exhaustion markers and the level of CD4^+^ T cell activation or ΔCD4 (Figures 4B,C).

### Secreting of IL-2 by CD4^+^ T Cells Was Associated With CD4^+^ T Cell Maintenance

**Figure 5:**
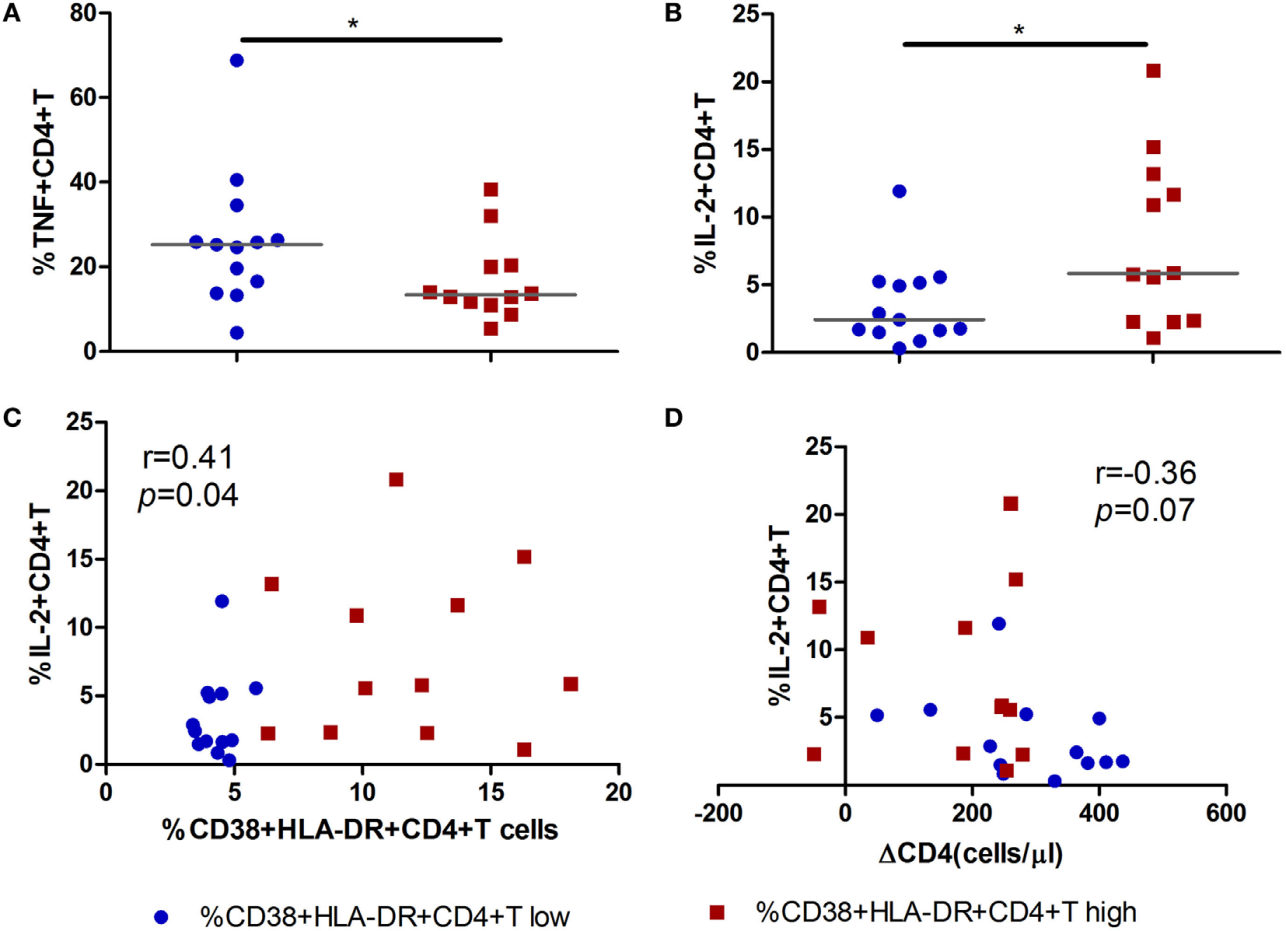
Secretion of IL-2 by CD4^+^ T cells in acute HIV-1 infection is associated with the maintenance of CD4^+^ T cells. Comparisons of TNF-α **(A)** and IL-2 **(B)** secreting capacity of CD4^+^ T cells between CD4^+^ T cell activation high (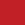) and low (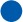) groups in acute HIV-1-infected patients. Correlation analysis between IL-2 and the level of CD4^+^ T cell activation **(C)** and ΔCD4 **(D)**. Significance of difference was calculated by Mann–Whitney *U* test. Correlation analysis was performed by Spearman’s rank test. Horizontal lines indicate median values (*p < 0.05, **p < 0.01, ***p < 0.001).

To further investigate the function of activated CD4^+^ T cells during acute infection stage and its role in CD4^+^ T cell decline over time, we analyzed the relationship between cytokine-producing (IFN-γ, TNF-α, and IL-2) CD4^+^ T cells and the level of CD4^+^ T cell immune activation. Intriguingly, decreased percentages of TNF-α-producing CD4^+^ T cells and increased percentages of IL-2-producing CD4^+^ T cells were found in the high immune activation group compared to the low activation group (all *p* < 0.05, Figures 5A,B). Correlation analysis revealed that increased IL-2 secreting capacity was positively correlated with the level of CD4^+^ T cell immune activation (*r* = 0.41, *p* = 0.04, Figure 5C) and negatively correlated with CD4^+^ T cell decline (ΔCD4, *r* = −0.36, *p* = 0.07, Figure 5D).

## Discussion

Several mechanisms are thought to contribute to CD4^+^ T cell depletion in HIV-1 infection. Of these, the two most acknowledged mechanisms are direct virus attack resulting in cytolytic effect and chronic immune activation leading to cell apoptosis (7–9, 20). Numerous reports have demonstrated that immune activation is a better predictor of clinical outcomes than plasma viral load in HIV-1-infected subjects (1, 2, 13, 21). Despite a general consensus on the association between chronic T cell activation and CD4^+^ T cell count and disease progression, whether immune activation during the acute phase results in CD4^+^ T cell depletion and immune suppression is unclear. In the present study, we reported a direct relationship between higher CD4^+^ T cell activation during acute HIV-1 infection and the less loss of CD4^+^ T cells after 2 years of acute infection (chronic infection stage). Activation of CD4^+^ T cells in early HIV-1 infection stage, therefore, may have a beneficial effect on CD4^+^ T cell homeostasis. Consistently, The Adult AIDS Clinical Trials Group (ACTG) interruption treatment trail found that higher baseline CD4^+^ T cell immune activation predicted the delay of viral rebound following treatment interruption (14). Ndhlovu et al. observed that the magnitude of CD8^+^ T cell activation and the rapidity to peak activation were inversely correlated with set point of viremia during hyperacute HIV-1 infection, indicating that CD8^+^ T cell responses are beneficial for subsequent immune control of acute infection (15). However, we did not find the similar results between CD8^+^ T cell activation and CD4^+^ T cell decline in the current study. These observations revealed that high levels of CD4^+^ T cell activation in acute HIV-1 infection represent a more competent immediate host immune response.

The ability of these cells to proliferate also correlates with CD4^+^ T cell decline. Ki67 expression is usually used as a surrogate marker for proliferation or even activation in chronic HIV-1 infection (9, 19). Here, we showed that in acute HIV-1 infection, the frequency of ki67 in CD4^+^ T cells correlated with the expression of phenotypic activation markers, and more importantly, it inversely correlated with CD4^+^ T cell decline in patients with acute infection over time. In addition, correlation between ki67 and CD4^+^ T cell decline was not observed in chronic stage patients (data not shown), suggesting that cycling may have a causal role in slow CD4^+^ T cell loss in acute HIV-1 infection. Ki67 protein is upregulated in all cell cycle phases except G0 (22). Previous studies in chronic HIV-1 infection have shown that ki67 may not really represent the cell cycle and turnover. Cycling CD4^+^ T cells are under cell cycle arrest (G1 phase or G1/S transition checkpoint), or die after entering S phase in chronically infected patients (23–25), suggesting that cell proliferation is not competent in CD4^+^ T cells in chronic infection. Taken together, we hypothesize that CD4 lymphocyte proliferation outcomes with acute and chronic infection are likely to be distinct. CD4^+^ T cell proliferation appears to be effective and beneficial in the acute phase, whereas in the chronic phase it is invalid or less effective. Therefore, ki67 is probably a better marker for CD4^+^ T cell decline in the setting of acute HIV-1 infection. In the absence of cell cycle arrest, CD4^+^ T cells are more competent to differentiate into functional subsets and compensate cell loss in AHIs.

The polyfunctional profile of CD4^+^ T cells was not significant different between high or low CD4^+^ T cell activation groups. However, higher fraction of IL-2-secreting CD4^+^ T cells in the high CD4^+^ T cell activation group was found in our study. Impaired IL-2-producing T cells was one of the first functional defects observed in chronically HIV-1-infected patients, which predicts loss of CD4^+^ T cells and progression to AIDS (26). Chronic immune activation prevents the induction of IL-2-expressing memory CD4^+^ T cells, and this associates with impaired function of HIV-1-specifc CD4^+^ T cells (27, 28). Although less well characterized than CD8^+^ T cell, HIV-1-specific CD4^+^ T cell proliferation and function during early HIV-infection seem to predict virus control and slower disease progression in the absence of therapy (29, 30). Taken together, these observations indicate that CD4^+^ T cell activation at the acute phase may have a crucial role in preventing CD4^+^ T cell depletion through production of cytokines and increased proliferation in AHIs. The IL-2 cytokine may provide important helper signals for later adaptive immune response.

Persistent antigen exposure and inflammatory status often leads to immune exhaustion, resulting in effector T cells progressively dysfunctional (31). A recent study reported that in primary HIV-1 infection, TIM-3^+^CD8^+^ T cells was associated with delayed progression (32). In our study, we found neither significant difference of exhaustion molecules between high and low CD4^+^ T cell activation groups nor the association between exhaustion molecules and the loss of CD4^+^ T cells. Our results may provide us evidence that less extent of T cell exhaustion was developed in acute HIV-1 infection.

Although high level of CD4^+^ T cell activation in early HIV-1 infection stage is beneficial for CD4^+^ T cell preservation, we proposed that this CD4^+^ T cell activation belongs to a homeostatic response to HIV-1. Excessive activation of CD4^+^ T cells may induce upregulation of CCR5 expression on CD4^+^ T cells, which increases targets for HIV infection and, therefore, may accelerate CD4^+^ T cell depletion (33, 34).

This study has several limitations. MSM cohort in our study was followed up every 2 months for acute infection screening. Most acutely infected patients were seroconversion in these 2 months and at late Fiebig stage. Therefore, it is interesting to know that whether the results differ from that in earlier AHI stage. In addition, our experiments were performed with PMA/Ionomycin T-cell stimulation, cytokine production should be determined upon HIV-specific antigen stimulation to further study the possible mechanisms that the preventing role of T cell activation in CD4^+^ T cell depletion during acute HIV-1 infection stage.

In summary, we found that higher level of CD4^+^ T cell immune activation in acute HIV-1 infection stage associates with subsequent slower CD4^+^ T cell decline in our study. Mechanism study revealed that greater levels of CD4^+^ T cell proliferation and IL-2 secretion under the high CD4^+^ T cell activation status may contribute to the CD4^+^ T cell preservation. Our research provides us a new insight into the role of T cell immune activation in acute HIV-1 infection in CD4^+^ T cell depletion over time.

## Ethics Statement

All HIV-1 infected participants provided written informed consents according to the declaration of Helsinki. All relevant experiments have been approved by the Beijing You’an Hospital Research Ethics Committee. The methods applied were carried out in accordance with the approved guidelines.

## Author Contributions

HX, XL, BS, and HW conceived the study and designed the experiments; HX, XL, XZ, LQ, and JS performed the experiments; HX, XL, XZ, and ZL analyzed the data; YZ and TZ contributed to reagents and materials; and HX, XL, WJ, and HW wrote the article. All authors read and approved the final manuscript.

## Conflict of Interest Statement

The authors declare that the research was conducted in the absence of any commercial or financial relationships that could be construed as a potential conflict of interest.
